# Supplementary material for: Handheld snapshot multi-spectral camera at tens-of-megapixel resolution
Source: Nat Commun. 2023 Aug 19;14:5043. doi: 10.1038/s41467-023-40739-3 (PMC10439928; doi:10.1038/s41467-023-40739-3)
Supplement: Supplementary file 1 — Supplementary Information [file 41467_2023_40739_MOESM1_ESM.pdf]

Supplementary Information for “Handheld Snapshot Multi-spectral Camera at Tens-of-Megapixel Resolution”

Weihang Zhang[1,†], Jinli Suo[1,2,3,†,*], Kaiming Dong[1], Lianglong Li[1], Xin Yuan[4], Chengquan Pei[5], and Qionghai Dai[1,2,*]

[1]Dept. of Automation, Tsinghua University, Beijing 100084, China
[2]Institute of Brain and Cognitive Sciences, Tsinghua University, Beijing 10008, China
[3]Shanghai Artificial Intelligence Laboratory, Shanghai 200232, China
[4]WestLake University, Hangzhou 310030, Zhejiang, China
[5]Xidian University, Xi'an 710071, Shaanxi, China
[†]These authors contributed equally to this work
[*]jlsuo@tsinghua.edu.cn; qhdai@tsinghua.edu.cn

Contents

1 Film making 2
2 Compact packaging of THETA 5
3 Encoded snapshot imaging model 5
4 Multi-spectral reconstruction algorithm 6
References 8

List of Figures

1 The optical path for producing wavelength dependent shifting using a dispersive plate glass . . . 2
2 The features of the wavelength-dependent codes by the film mask . . . 3
3 The system for generating the coded film . . . 4
4 The adopted fiber optic plate (FOP) and its performance in tens-megapixel image transmission . . . 6
5 The structure of the adopted coarse-to-fine network for multi-spectral reconstruction . . . 7
6 Multi-spectral reconstruction of a complex natural scene with different growing plants . . . 8

For our high throughput compact computational spectral camera THETA, the fabrication of the encoding mask, high precision packaging of the prototype, and computational reconstruction algorithm are all of vital importance for the final performance. Here we provide the details of key optical encoding and computational decoding techniques.

1 Film making

Basic idea. In this paper we propose a method engineering a tens-of-megapixel multi-spectral encoding mask, to circumvent the limited pixel count of commercial programmable spatial light modulators and incapability of photolithographic mask to conduct spectrum-specific modulation. Inspired by the fact that shifting a random 2D pattern laterally can produce a group of approximately independent random patterns, here we design a film imaging system to introduce spectrum-dependent shifts to a high resolution binary random pattern and record the superimposition of the shifted patterns onto the film. The generated film acts as a multi-spectral encoding mask and is attached to the sensor to implement single-shot spatio-spectral encoding of the scene.

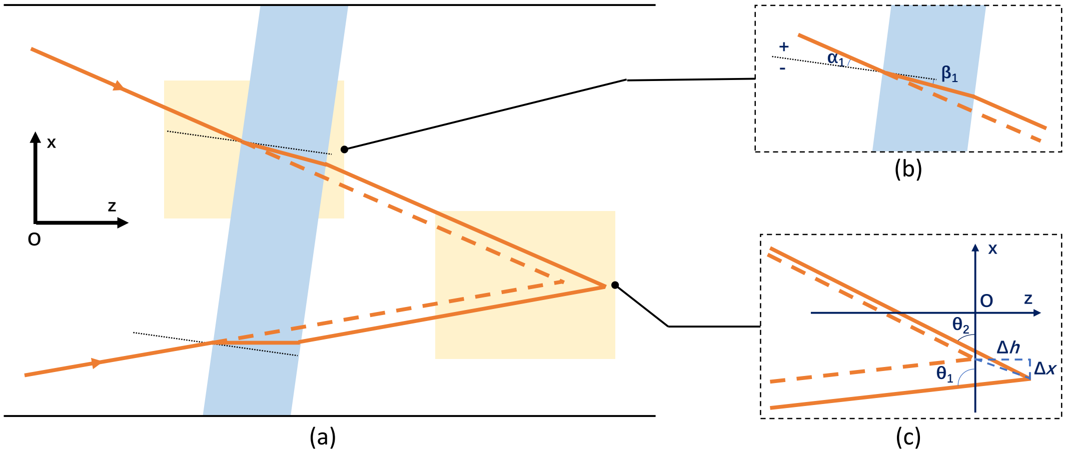

Supplementary Figure 1. The optical path for producing wavelength dependent shifting using a dispersive plate glass (blue bar), with the dashed and solid lines for the light rays before and after introducing the disperser. (a) The cross section view of the light transmission. (b) The zoomed-in view of one local region detailing the effect of dispersive plate glass. (c) A diagram illustrating the lateral (Δx) and axial (Δh) shift on the sensor, where θ_1 and θ_2 are respectively the intersecting angles of the two orange rays with respect to the positive direction of x-axis of the sensor.

Geometric model. In implementation, the spectrum-dependent shifting is generated by inserting an inclined planar dispersion component—plate glass with low Abbe number, i.e., the refractive index changes significantly with the incident wavelength—between the lens and the focal plane, as illustrated in Supplementary Figure 1(a). When the incident light travels obliquely at a certain angle to the plate glass, the outgoing light ray of each wavelength is shifted by a mount determined by its refraction index, as shown in Supplementary Figure 1(b). The shifts at different wavelengths can be calculated explicitly from the diagram in Supplementary Figure 1(c), in which we denote the aperture size as F, aperture-to-sensor distance as L, and set the coordinate origin at the center of the sensor. The two rays that respectively pass through the upper and lower boundary of the aperture will converge at coordinates $(x, 0)$ on the sensor plane, and their intersecting angles with the positive direction of x-axis of the sensor are respectively denoted as θ_1 and θ_2, with

$$\tan\theta_1 = -\frac{L}{\frac{F}{2}+x} \tag{1}$$

and

$$\tan\theta_2 = \frac{L}{\frac{F}{2}-x}. \tag{2}$$

As shown by the blue bar in Supplementary Figure 1, here we suppose that the plate glass is with thickness d, the refractive index at the wavelength λ is n_λ, and keep an intersecting angle θ with the x-axis of the sensor plane. Defining the angle between a ray above the normal and the normal as positive, and denoting incident angles of the two rays are respectively $\alpha_j (j = 1, 2)$, it can be derived that

$$\alpha_j = \theta - \theta_j, \tag{3}$$

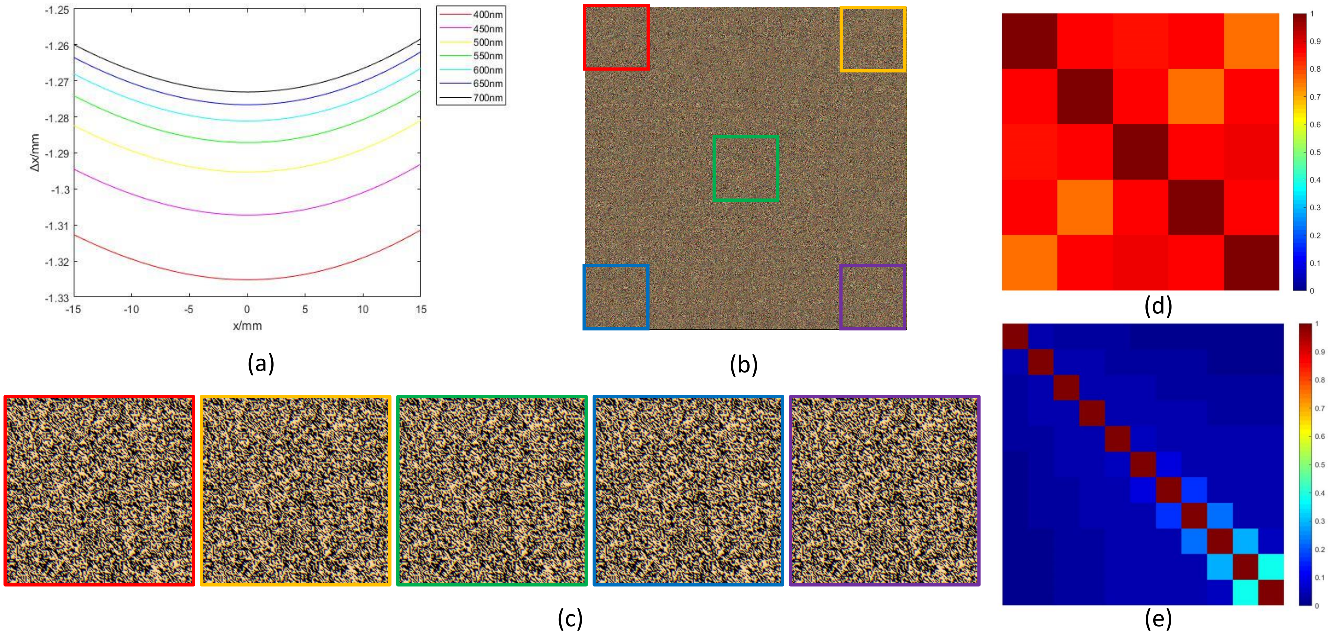

Supplementary Figure 2. The features of the wavelength-dependent codes by the film mask. (a) The relationship between the x-axis coordinate on the sensor and the displacement caused by the disperser at different wavelengths. (b) The RGB image of the simulated 65-megapixel encoding mask, which consists of an overlay of displaced masks over a broad range of wavelengths. (c) Zoomed-in views of the simulated encoding masks at five locations marked with different colors in (b). (d) Plot of the correlation coefficients among the simulated masks in (c), averaged over 11 wavelengths (from 400nm to 700nm, with an interval of 30nm). (e) Correlation coefficients among the encoding masks at 11 wavelengths.

and the exit angles are

$$\beta_j = -\alpha_j / n_\lambda. \tag{4}$$

According to the geometry, it can be derived that the offset of the two rays from the original position along the x-direction of the sensor is

$$\Delta x_j = \frac{d\,(\tan\beta_j + \tan\alpha_j)\cos\alpha_j}{\sin\theta_j}. \tag{5}$$

The position where the two rays reconverge has an offset in both the x-direction and the z-direction from the previous convergence point. The offset in the x-direction is

$$\Delta x = \Delta x_1 - \frac{L}{F\tan\theta_1}(\Delta x_2 - \Delta x_1) = \Delta x_2 - \frac{L}{F\tan\theta_2}(\Delta x_2 - \Delta x_1) \tag{6}$$

i.e.,

$$\Delta x = \Delta x_1 + \frac{F+2x}{2F}(\Delta x_2 - \Delta x_1) = \Delta x_2 - \frac{F-2x}{2F}(\Delta x_2 - \Delta x_1), \tag{7}$$

and the offset along the z- axis is

$$\Delta h = \frac{L}{F}(\Delta x_2 - \Delta x_1). \tag{8}$$

In implementation, considering that it is difficult to insert an optical element between the lens and the sensor of a commercial film camera, we achieve similar shifting by placing the planar disperser between the lens and the lithographic mask. In this case, we multiply the offset Δx by a factor L/D, where D is the distance between the aperture and the lithographic mask.

We derive the relationship between the offsets of varying wavelengths at different positions, as plotted in Supplementary Figure 2(a). One can see that the disperser introduces larger offsets at short wavelengths and the offsets are symmetric laterally. More importantly, the among-wavelength difference of the set is consistent at different positions, which helps to generate well structured encoding patterns together with the periodic binary pattern on the lithography mask. To test the repeatability of the binary repetitive coding patterns after applying shifting, we select five patches highlighted in Supplementary Figure 2(b) and (c)—a central region and its repetitive counterparts (with the highest similarity) located in four corner ones—from the

sensor mask and plot their correlation in Supplementary Figure 2(d). The results show that there exist high similarity among the patches in different regions. The plot consists with the observation in Supplementary Figure 2(a) that the variation of the offsets at different positions is small under the same wavelength. We also test the independence of the coding patterns at different spectral bands, and the results in Supplementary Figure 2(e) show that the coding patterns of different bands are highly independent from each other.

In sum, the coding mask is highly repetitive across the camera sensor to facilitate training reconstruction algorithms working for large scale data, and distinctive across spectral channels to ensure high reconstruction quality.

Setup for fabrication. Under this mechanism, we take a plate glass photo-etched with binary random patterns as object and use the Mamiya RB 67 medium format film camera to superimpose its spectrally-shifted versions onto a Fuji PROVIA 120 film. The top-left inset in Supplementary Figure 3 illustrates the superimposition of binary masks with spectrum dependent shifts. The specific experimental setup aims to ensure that the lateral offset of the calibrated mask between adjacent spectral channels should not be less than the size of one sensor pixel ($3.2\mu m$), thus achieving effective encoding. According to Supplementary Figure 2, the shifts between adjacent spectral channels are distinguished: the larger the wavelength, the smaller the shift. Therefore, we only consider the displacement between the two spectral channels with long wavelengths, and it depends on the object distance of the film photography as well as the Abbe number, the angle to the optical axis, and the thickness of the dispersion element, but is independent of the aperture size. As verified by the derivation in the previous section and the provided simulation program, the smaller the object distance, the larger the offset. However, an object distance below the minimum working distance of the film camera may lead to image distortion. Therefore, the minimum working distance of $300mm$ is adopted for the object distance, and in this case, the magnification of film photography is around $0.83\times$. Furthermore, in order to match the size of the mask unit and sensor pixel, the size of the binary mask unit is $3.84\mu m$. Moreover, the thicker the dispersion element, the larger the offset, but at the same time the image quality on the film is degraded due to internal scattering and increased axial displacement among channels. Finally, the angle between the dispersion element and the optical axis also exhibits a nonlinear relationship with the offset, which reaches its peak at the angle about 40–45 degrees. Therefore, we have adopted a type of dispersion glass with the lowest Abbe number in Schott's product list, with a thickness of $7mm$ to keep the variation of axial displacement within 1 mm and an angle with the optical axis of about 40 degrees. According to our derivation under the aforementioned experimental setup, a lateral offset of about $3.2\mu m$ can be generated on the image plane between the calibrated masks of the spectral channels with center wavelengths of $640nm$ and $660nm$, respectively.

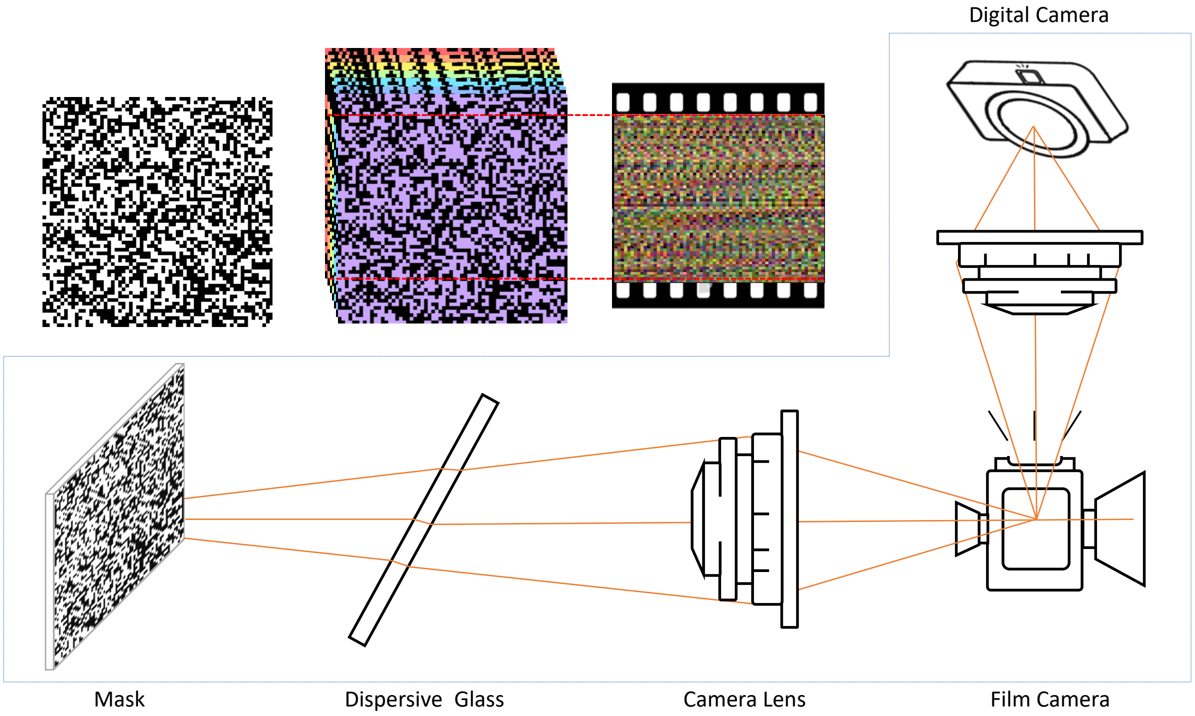

Supplementary Figure 3. The system for generating the coded film.

We use three more strategies for high quality film fabrication. (i) The lithographic pattern is an array repetition of an 256×256 random 0-1 entries. Such repetitive layout can produce similar spectral coding across the sensor and facilitate training

deep neural networks that decode multi-spectral data cube from the snapshot. (ii) The film making process demands precise and accurate control of focusing and exposure time but it is difficult to conduct such high precision setting from the viewfinder of a film camera. To address this issue, we build a vertical auxiliary arm to capture the image of the viewfinder with an digital industrial camera, which produces consistent images on the film, viewfinder, and digital camera sensor. One can judge the expected quality of film and fine tune the setting of the film camera by observing the readout of the industrial camera. (iii) A fiber-coupled xenon light source (CME-303 solar simulator manufactured by Microenerg) is used to provide collimated illumination with approximately flat spectrum. Supplementary Figure 3 shows the whole light path.

2 Compact packaging of THETA

It is non-trivial to conduct pixel-wise encoding using the generated film mask on a high SBP imaging setup. An intuitive way is to place the coding mask at the image plane and relay it onto the sensor plane using a relay lens. Nevertheless, a high SBP relay lens with ten-megapixel resolution is bulky and suffers from severe vignetting artifacts for large field-of-view imaging. Directly attaching the film onto the sensor plane is infeasible either, because expertise demanding mechanical engineering is required to remove the protection glass and attach the film flatly without pattern distortion. In addition, possible dust might cause dead spots and the film cannot be replaced easily once packaged. To circumvent this challenge, we introduce a fiber optic plate (FOP, an image-preserving fiberoptic bundle) to transmit the image from its front face (at the image plane) to the rear face (at the sensor plane), i.e., couple the encoded image directly onto the sensor, at high transmission efficiency. The zoomed-in view of the FOP is shown in Supplementary Figure 4(a), captured under a Nikon 40× microscope.

In our implementation, we use a cover-opening version IDG-6500-M-G-CXP6 sensor and package a $31\times23\times9mm$ FOP onto the surface of the bare sensor. The thickness of the FOP is designed to be slightly above the surface of the sensor chip, which simplifies the mounting of the coding film. Notably, the packaging is carried out in a dust-free environment. The mechanical structure, front view and the corresponding section view of the packaged camera are illustrated in Supplementary Figure 4(b), where a piece of customized cover glass is added for fixing the mask.

After FOP packaging, we mount a BV-L1020 lens onto the IDG-6500-M-G-CXP6 camera sensor by M58 thread. Considering the shifting of imaging plane introduced by the FOP, the sensor is moved back by around $9mm$ to obtain the sharp encoded measurement, i.e., about $9mm$ of thread is exposed between the lens and the camera. Besides, we measure the transmission spectra of the blank film and install a filter with customized spectral profile roughly compensating the film's imbalanced transmission. The final packaged imaging setup is of similar appearance to a commercial camera. We verify the light efficiency and detail preservation of the FOP on image transmission. Here we use a the resolution target (USAF) as the target object and compare the imaging results of the original camera and packaged camera with the same lens. The comparison is shown in Supplementary Figure 4(c) and (d), which tell that the FOP is of high transmission efficiency and can preserve the details at high precision, which is an ideal option for relaying high SBP images in a compact manner.

3 Encoded snapshot imaging model

Let us denote the image of the binary mask etched on the lithographic plate as $\mathbf{M}(x,y)$, the coding pattern on the sensor is the superimposition of $\mathbf{M}$'s spectrally shifted versions. For the coordinate (x,y) on the sensor, according to Eq. 7, we can explicitly obtain the shift/offset generated at wavelength λ as $(\Delta_x(x,\lambda),\Delta_y(y,\lambda))$. Given the spectral radiance of the broadband light source used for coding film fabrication $\mathbf{l}_0(\lambda)$ and the spectral transmittance of the blank film $\mathbf{t}_{\text{film}}(\lambda)$, the coding at coordinate (x,y) of the packaged sensor (i.e., the transmittance of the film mounted on the sensor) is

$$\mathbf{C}(x,y,\lambda) = \mathbf{M}\left(x-\Delta_x\left(x_0,\lambda\right), y-\Delta_y\left(y_0,\lambda\right)\right)\mathbf{l}_0(\lambda)\mathbf{t}_{\text{film}}(\lambda). \tag{9}$$

with (x_0,y_0) on the binary pattern derived by

$$x_0+\Delta_x\left(x_0,\lambda\right) = x, \tag{10}$$
$$y_0+\Delta_y\left(y_0,\lambda\right) = y. \tag{11}$$

Given a specific scene with intensity distribution $\mathbf{S}(x,y,\lambda)$, its encoded measurement is derived by

$$\mathbf{I}(x,y) = \int_{\lambda_1}^{\lambda_2} \mathbf{l}_1(\lambda)\cdot\mathbf{S}(x,y,\lambda)\cdot\mathbf{t}_{FOP}(\lambda)\mathbf{t}_{\text{rec}}(\lambda)\cdot\mathbf{C}(x,y,\lambda)d\lambda, \tag{12}$$

where $[\lambda_1,\lambda_2]$ is the range of film's response spectrum, $\mathbf{l}_1(\lambda)$ is the spectrum of environmental illumination that is assumed to be spatially uniform, $\mathbf{t}_{\text{FOP}}(\lambda)$ is the intrinsic transmission spectrum of the FOP, and $\mathbf{t}_{\text{rec}}(\lambda)$ is the "reciprocal" transmission curve of blank film to correct the unbalanced spectrum of the film.

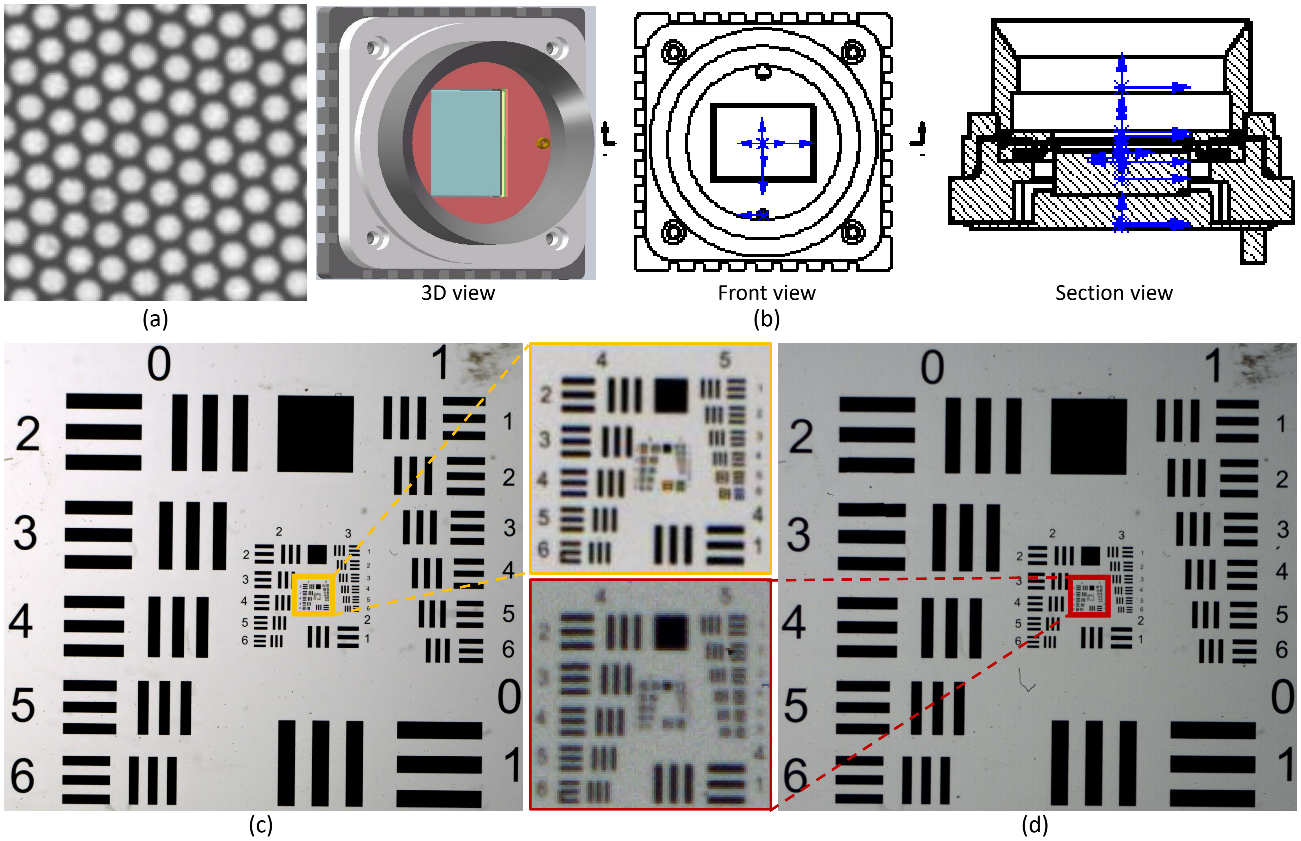

Supplementary Figure 4. The adopted fiber optic plate (FOP) and its performance in tens-megapixel image transmission. (a) The magnified view of the FOP captured by 40× objective lens. (b) The mechanical structure of the proposed camera, where the blue area is the FOP packaged on the sensor, and the pink area is the cover glass customized for fixing the mask. (c) The USAF image captured by the original camera without FOP, and the zoomed-in image of the central region. (d) The USAF image captured by the proposed camera packaged with FOP, and the zoomed-in image of the same region with (c). Note that the sensor package with FOP is displaced by around 9mm for refocusing.

It is worth noting that, the encoding of our approach is different from conventional CASSI[1]. CASSI encodes the spectrally shifted data cube with a random mask, while the proposed design multiplexes the spectrally aligned data cube with a group of shifted color masks. Therefore, we need slight changes in the data processing stage when designing or applying reconstruction algorithms.

4 Multi-spectral reconstruction algorithm

For the reconstruction of multi-spectral data at such large scale, the efficiencies in both training and inference stages are of crucial importance. Fortunately, the proposed encoding mask can be well structured, with a approximately repetitive layout. In our implementation, the whole pattern consists of 27×37 slightly overlapping blocks (around 1000 blocks in total), each with 256×256 pixels. The encoding patterns in nearby blocks are of quite high similarity and can share a common deep neural network for high quality reconstruction, but it is impractical to learn a single network working well for all the blocks since the difference in far apart encoding patterns are non-negligible. As a trade-off, we propose to divide the blocks into groups and learn their respective reconstruction networks. Benefiting from the repetitive code design, we can train the reconstruction networks in a coarse-to-fine manner (learn a base model and then adapt them fast to data in different groups) instead of learning each model from scratch, which significantly shortens the training time.

For each group of blocks, considering of both high reconstruction quality and fast inference, we employ a recently proposed network with state-of-the-art performance, designed with a sparse Transformer structure[2]. Suppose the image size is $H \times W$ and there are Λ non-overlapping spectral channels, the network design and working flow is illustrated in Supplementary Figure 5. First, the encoded measurement $\mathbf{I} \in \mathbb{R}^{H \times W}$ is taken as an initial estimation of each spectral channel, i.e., the spectral data cube

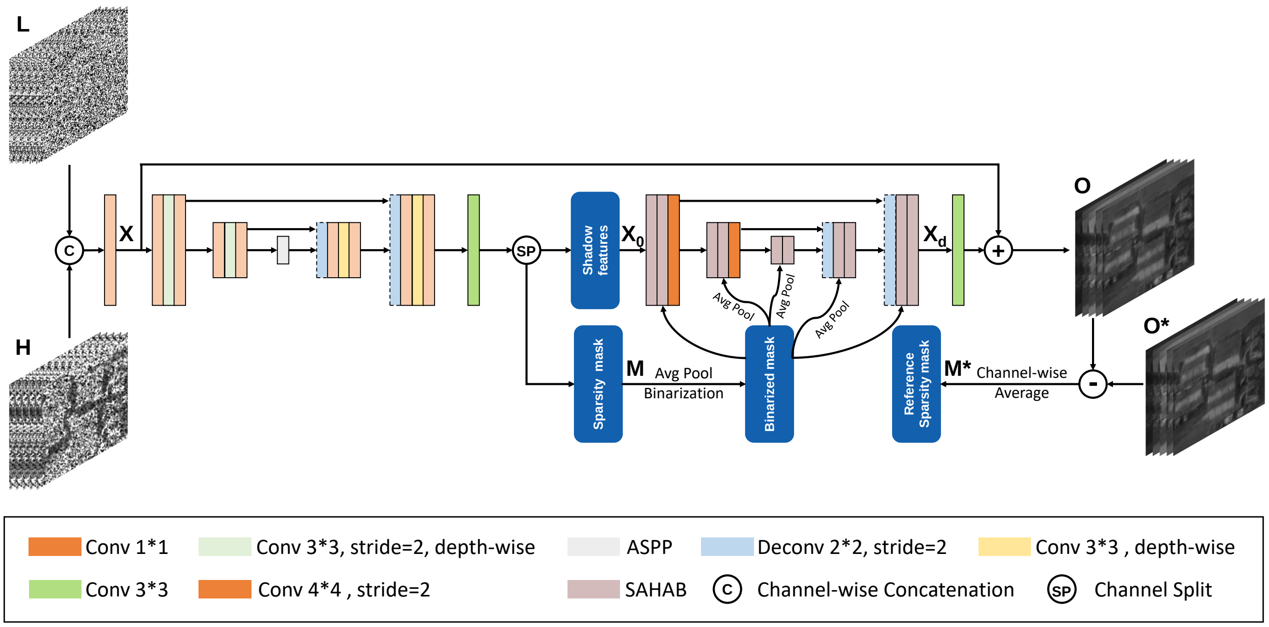

Supplementary Figure 5. The structure of the adopted coarse-to-fine network for multi-spectral reconstruction. An image patch from Figure 1 is used as an exemplar input.

$\mathbf{H} \in \mathbb{R}^{H \times W \times \Lambda}$ is initialized as

$$\mathbf{H}(x,y,n_\lambda) = \mathbf{I}(x,y), \tag{13}$$

where $n_\lambda \in [1,\ldots,\Lambda]$. Then $\mathbf{H}$ is concatenated with the acquired multi-spectral mask $\mathbf{C}$, generating the Λ-layer initialized features $\mathbf{X} \in \mathbb{R}^{H \times W \times \Lambda}$ after passing a convolutional layer with kernel size 1×1. Next, $\mathbf{X}$ is processed by a sparsity estimator which has a U-Net structure with skip connection and Atrous Spatial Pyramid Pooling (ASPP) module[3]. The U-net provides a $(K+1)$-layer output, containing a sparsity mask $\mathbf{M} \in \mathbb{R}^{H \times W}$ and the shallow features $\mathbf{X_0} \in \mathbb{R}^{H \times W \times K}$. The latter passes another U-Net structure characterized by the spectra-aggregation hashing multi-head self-attention mechanism to embed deep feature $\mathbf{X_d} \in \mathbb{R}^{H \times W \times K}$, which then passes a convolutional layer with kernel size 3×3 to reconstruct the residue between the input multi-spectral image and the ground truth. Finally, the reconstructed multi-spectral image $\mathbf{O} \in \mathbb{R}^{H \times W \times \Lambda}$ is obtained by adding up the residue and the initialized features $\mathbf{X}$.

For the sparsity estimator, we take the average reconstruction residue over all the color channels as a reference

$$\mathbf{M}^* = \frac{1}{\Lambda} \sum_{n_\lambda=1}^{N} |\mathbf{S}(:,:,n_\lambda) - \mathbf{S}^*(:,:,n_\lambda)|, \tag{14}$$

in which large values represent dense spectral information that is difficult to retrieve, thus providing supervision over the output sparsity. The loss function for network training is defined of weighted summation of two items, including the difference between the multi-spectral image $\mathbf{S}$ and the ground truth $\mathbf{S}^*$, and the difference between the mask output $\mathbf{M}$ by the sparse estimator and the reference sparsity mask $\mathbf{M}^*$:

$$\mathscr{L} = \mathscr{L}_2 + \mu \cdot \mathscr{L}_s = ||\mathbf{S} - \mathbf{S}^*||_2 + \mu \cdot ||\mathbf{M} - \mathbf{M}^*||_2, \tag{15}$$

with μ being the weighting factor balancing two items.

For the reconstruction of the residue, the algorithm borrows the multi-head self-attention mechanism in the transformer model[4]. Specifically, the object of the proposed spectra-aware hashing attention block (SAHAB) is the vectors in a bucket which is formed by sorting and splitting the feature vectors by their value from hash mapping, and the feature vectors are previously filtered by the sparsity mask with a threshold to only reserve the pixels with dense information. According to the transformer mechanism, for the query vector $\mathbf{q} \in \mathbb{R}^{C \times 1}$ in a bucket $\mathbf{B}_i$. The output of the multi-head self-attention module is[2]:

$$\text{SAH-MSA}(q,\mathbf{B}_i) = \sum_{n=1}^{N} \mathbf{W}_n \sum_{k \in \mathbf{B}_i} [\underset{k_0 \in \mathbf{B}_i}{\text{softmax}}(\frac{q^T \mathbf{U}_n^T \mathbf{V}_n k_0}{\sqrt{d}})] \mathbf{W'}_n k, \tag{16}$$

Where $\mathbf{U}_n$, $\mathbf{V}_n$ and $\mathbf{W'}_n \in \mathbb{R}^{d\times C}$ are the query, key and value matrix respectively, and $\mathbf{W}_n \in \mathbb{R}^{C\times d}$ is the weight matrix of the attention head. Multiple rounds of hashing are adopted to further optimize the element clustering, i.e., the multi-round output is the weighted sum of each single-round output. For each query element $\mathbf{q}$, the weight of each round is the proportion of the sum of its score vectors of this round in the sum of those of all rounds, indicating the similarity between $\mathbf{q}$ and all elements in the belonged bucket in this round.

To demonstrate the advantageous spatial and spectral resolution of the final reconstruction, especially for the scenes containing rich texture or complex features, we further conduct the multi-spectral reconstruction for a large-FOV outdoor scene containing various growing plants, with the results shown in Supplementary Figure 6. In the top row, we display the encoded measurement, retrieved spectral channels, and the synthesized RGB image, while in the middle and bottom rows the zoomed-in view of two representative local regions containing rich high frequency features, including leaf veins and petal edges.

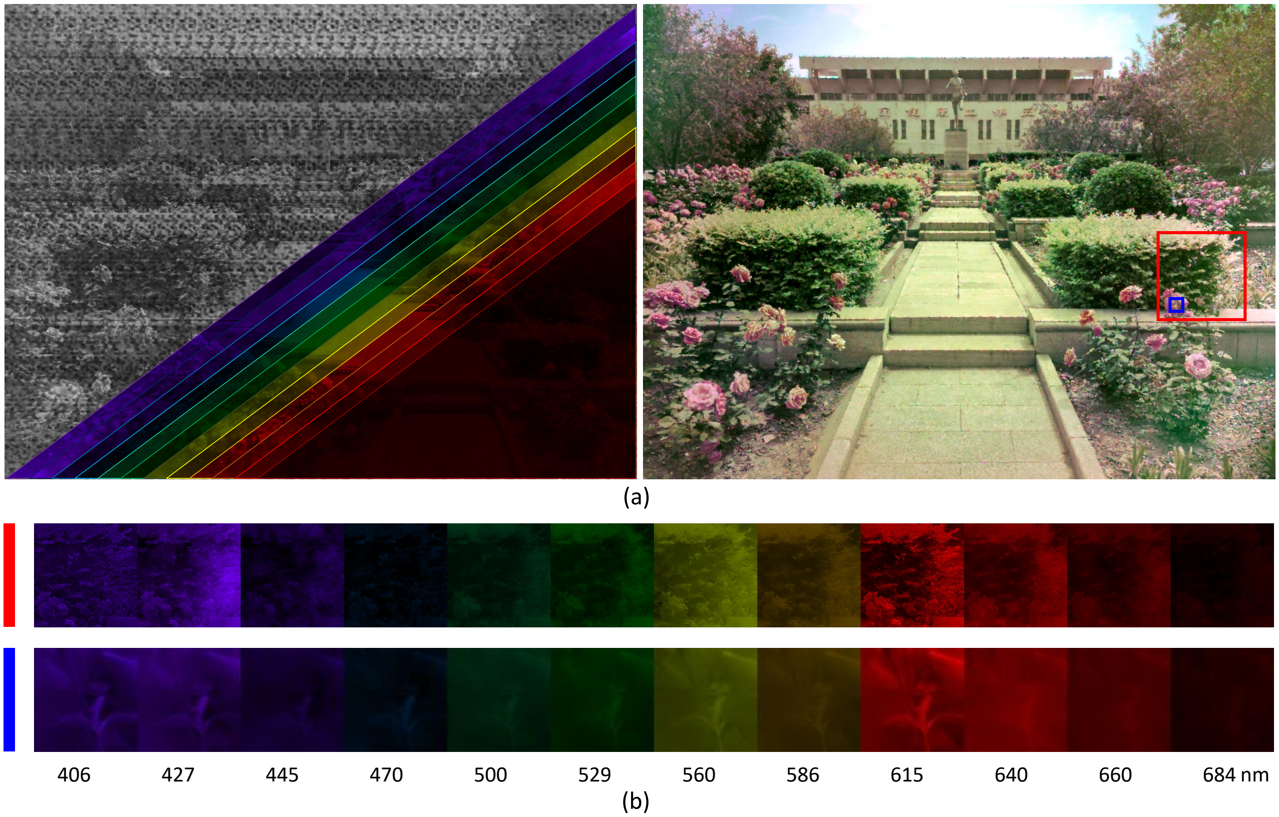

Supplementary Figure 6. Multi-spectral reconstruction of a complex natural scene with different growing plants. (a) The coded measured and multi-spectral images (left) and the synthesized RGB view (right). (b) The multispectral reconstruction in the visible region highlighted with the red and blue boxes in (a), colored by the RGB value of the corresponding wavelength.

References

1. Wagadarikar, A., John, R., Willett, R. & Brady, D. Single disperser design for coded aperture snapshot spectral imaging. *Appl. Opt.* **47**, B44–B51 (2008).

2. Lin, J. *et al.* Coarse-to-fine sparse transformer for hyperspectral image reconstruction. In *European Conference on Computer Vision*, 686–704 (Springer Nature Switzerland, 2022).

3. Chen, L.-C., Papandreou, G., Schroff, F. & Adam, H. Rethinking atrous convolution for semantic image segmentation. *arXiv preprint arXiv:1706.05587* (2017).

4. Vaswani, A. *et al.* Attention is all you need. *Adv. Neural Inf. Process. Syst.* **30** (2017).
