# Supplementary material for: Handheld snapshot multi-spectral camera at tens-of-megapixel resolution
Source: Nat Commun. 2023 Aug 19;14:5043. doi: 10.1038/s41467-023-40739-3 (PMC10439928; doi:10.1038/s41467-023-40739-3)
Supplement: Supplementary file 3 — Description of additional supplementary files [file 41467_2023_40739_MOESM3_ESM.docx]

**Description of Additional Supplementary Files Document**

**Video - Supplementary Movie 1**

The RGB time-lapse video synthesized from the 12 reconstructed spectral channels of the diffusion process of sewage in a miniature landscape of a river.

**Video - Supplementary Movie 2**

The dynamic distribution of two kinds of pollutants in the river landscape represented by two pseudo-colors (blue and green), generated according to the multi-spectral time-lapse data of 12 reconstructed channels. The darker the color, the higher the concentration.
